# A longitudinal investigation of the impact of typology of urinary incontinence on quality of life during midlife: Results from a British prospective study

**DOI:** 10.1016/j.maturitas.2009.09.015

**Published:** 2009-12-20

**Authors:** Gita D. Mishra, Tim Croudace, Linda Cardozo, Diana Kuh

**Affiliations:** a MRC unit for Lifelong Health and Ageing, University College and Royal Free Medical School, 33 Bedford Place, London WC1B 5JU, United Kingdom; b Department of Psychiatry, University of Cambridge, Box 189, Level E4, Addenbrooke's Hospital, Hills Road, Cambridge CB2 2QQ, United Kingdom; c Kings College London, Denmark Hill, London SE5 9RS, United Kingdom

**Keywords:** Urinary incontinence symptoms, Menopause, Quality of life, Latent class analysis

## Abstract

Using prospective data from 983 British women born in 1946, the study aims to describe the profiles of symptoms of stress, urge, and severe incontinence, and to relate these to change in quality of life. Based on the longitudinal patterns of symptoms experienced, four groups of women were defined: ‘low symptom’, ‘onset’, ‘recovering’, and ‘chronic’. Childhood enuresis was associated with being in the ‘chronic’ group for urge and severe incontinence. Women in the ‘recovering’ group for stress incontinence experienced an improvement in the physical health domain (regression coefficient (95% CI): 0.1(0.02, 0.18)) compared with women without symptoms. This relationship existed beyond the effects of ageing, menopausal status, current life stress, and reproductive, lifestyle, and social factors. More research is needed to understand the mechanism that link childhood enuresis to being in the ‘chronic’ group for urge and severe incontinence.

## 1 Introduction

Urinary incontinence adversely impacts upon quality of life (QoL) and participation in everyday activities [1]. It is associated with poor self-rated health and depressive symptoms [2]. Longitudinal information on symptoms associated with each type of incontinence may be used to classify women into clinically meaningful subgroups. To our knowledge, there are only a few longitudinal studies of urinary incontinence [3,4] and none have taken a typological approach to the pattern of evolution or remission of urinary symptoms.

The aims were to: a) characterise the patterns of change or stability of symptoms of stress, urge, and severe incontinence and b) to determine whether these patterns were related to change in QoL during midlife while taking into account the effects of ageing, menopausal status, current life stress, and reproductive, lifestyle, and social factors.

## 2 Methods

### 2.1 Study design, materials and methods

The MRC NSHD is a nationally representative sample of 2547 women and 2815 men followed up regularly since their birth in March [5]. This paper draws upon data collected annually from women cohort members who provided information on the symptoms of stress, urge, and severe incontinence from ages 48 to 54 years [6]. A total of 983 (64%) women provided complete information on the QoL items and all relevant risk factors/confounders collected across the life course.

### 2.2 Incontinence and other urinary symptoms

Each year, women who replied positively to the question “Do you ever lose any urine when you cough, sneeze, laugh, run or exercise?” were classified as having symptoms of stress incontinence (hereafter referred to as just stress incontinence). Those who replied positively both to the question “Do you ever have an urgent and strong desire to pass urine which is difficult to control?” and to the follow up question “Do you ever lose any urine before you reach the toilet?” were classified as having the symptoms of urgency and urge incontinence (hereafter referred to as urge incontinence). Severe incontinence was defined as involuntary loss occurring twice a month or more over the previous year and the reported loss of more than a few drops of urine.

### 2.3 Change in quality of life

Following factor analysis of 11 questionnaire items for perceived change, four quality of life domains were obtained: physical health (physical health, energy level, body weight), psychosomatic status (nervous and emotional state, self-confidence, ability to concentrate), personal life (family life and time for self, hobbies, and interests), and sex life [7]. An additional item on reported ‘difficulties during intercourse’ was also used.

### 2.4 Other risk factors or potential confounding factors

Childhood enuresis was defined from maternal reports of bedwetting ‘occasionally’ or at least ‘several nights a week’ or wetting ‘sometimes’ during the day when study members were 6 years old [6]. Menopausal status [8] and current life stress were assessed at each age from 48 to 54 years. Reproductive, lifestyle, and social factors were obtained at age 43.

### 2.5 Statistical analysis

Three statistical models were used in the following sequence: a) longitudinal latent class analysis [9], on each of the symptoms of stress, urge, and severe incontinence, assessed over 7 consecutive years, to identify women with different patterns of the symptoms; b) Chi square analysis to test the association between factors that may be associated with urinary incontinence; and c) Generalised Estimating Equations (GEEs) to examine the associations between these patterns on the change in QoL, while adjusting for risk or confounding factors.

## 3 Results

**Fig. 1:**
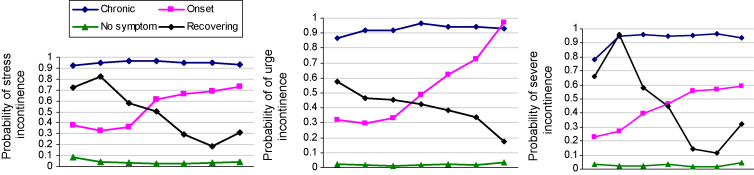
Profiles of urinary symptoms reported annually from age 48 to 54 years (*x*-axis).

The figure shows four groups of women based on the longitudinal patterns of the symptoms experienced. The clusters can be described as ‘low symptom’, ‘onset’, ‘recovering’, and ‘chronic’ for stress (*n* = 432, 123, 58, 370 women), urge incontinence (*n* = 680, 90, 133, 80 women) and severe incontinence (*n* = 615, 166, 31, 171 women) (see Fig. 1).

### 3.1 Patterns of urinary symptoms

Compared with the ‘low symptom’ group, women in the ‘onset’ group for stress incontinence had higher number of GP visits (37% vs. 30%), had more vaginal delivery (81% vs. 78%) and were more inactive (62% vs. 58%). A higher percentage of women in the ‘chronic’ group’ for urge incontinence had experienced childhood enuresis (17% vs. 10%), while more women in the ‘onset’ group visited their GP (42% vs. 33%). Similarly more women in the ‘chronic’ group for severe incontinence had experienced childhood enuresis (14% vs. 11%), and had not attained university or higher degree qualifications (30% vs. 38%).

### 3.2 QoL

For each year of the study, 50–60% women recorded deterioration in physical health, while 20% reported a decline in the sex life domain. Results from GEEs revealed that women in the ‘recovering’ group for stress incontinence experienced an improvement in the physical health domain (adjusted regression coefficient (95% confidence interval): 0.1 (0.02, 0.18)) compared with women without symptoms. Women in the ‘chronic’ group for severe incontinence were less likely to report difficulty with intercourse (−0.6 (−1.03, −0.1)). No other relationship was detected between patterns of symptoms and QoL.

## 4 Discussion

This study is the first to use prospective data collected annually, over 7 consecutive years, to describe the patterns of change or stability (typology) of the symptoms of stress, urge, and severe incontinence in midlife and to relate them changes in quality of life.

The association found between childhood enuresis and being in the ‘chronic’ group for the symptoms of urge/severe incontinence in midlife suggests that they have a common aetiology [6]. Women who were in the ‘onset’ group for stress or urge incontinence had higher GP consultation at age 43, five years prior to the reported urinary symptoms, suggesting that these women may already be in poorer health.

Profiles of stress and severe incontinence were associated with two of the four domains of the reported change in QoL. An improvement in the physical health domain for women in the ‘recovering’ group for the symptoms of stress incontinence suggests that QoL can be improved by managing their urinary symptoms. Women in the chronic group for severe incontinence were less likely to report difficulty with intercourse as it may be that they have found a way to adapt to their symptoms. Other women, with no change in QoL, may have become used to their symptoms or found an effective management strategy.

One limitation of the study is that the QoL items have not been validated externally. Nevertheless, we have previously demonstrated the QoL domains were associated with a range of psychosocial and biological factors [7].

### 4.1 Concluding message

Longitudinal information on symptoms associated with each of the incontinence type may be used to classify women into clinically meaningful subgroups. Childhood enuresis was associated with being in the ‘chronic’ group for urge and severe incontinence in midlife. Compared with women who had ‘low symptoms’, women recovering from stress incontinence were more likely to experience an improvement in their physical health.

## Funding

The Wellcome Trust Grant provided financial support for GDM. The Medical Research Council provided funding for the National Survey of Health and Development and financial support for DK.

## Competing interests

None declared.

## Ethical approval

North Thames Multicentre Research Ethics Committee.
